# Obstetric fistula awareness in The Gambia, Côte d’Ivoire, Madagascar and Sierra Leone: A cross-sectional analysis of Demographic and Health Surveys data

**DOI:** 10.4102/jphia.v17i1.1531

**Published:** 2026-02-17

**Authors:** Hayley Pierce, Emily Leclerc, Nicole Peterson

**Affiliations:** 1 Department of Sociology, Brigham Young University, Provo, United States of America

**Keywords:** obstetric fistulae, education, Africa, health awareness, women’s empowerment

## Abstract

**Background:**

Obstetric fistulas represent a frequently overlooked consequence of inadequate maternal health care and are largely preventable and treatable, with fistula awareness playing a key role in these efforts. This article aimed to understand the levels of fistula awareness and socioeconomic factors that aid or hinder that awareness in four African countries.

**Aim:**

The intent was to increase knowledge of the importance of fistula awareness and propose possible pathways to further increase this awareness in the region.

**Setting:**

This research used cross-sectional data from the 2019–2021 Demographic and Health Surveys (DHS) in The Gambia, Côte d’Ivoire, Madagascar and Sierra Leone.

**Methods:**

We noted the prevalence of fistulas in these populations and used weighted logistic regression (*n* = 60 128) to examine the relationships between key factors and fistula awareness overall and by country.

**Results:**

Overall, 0.01% of respondents report having a fistula, and 33% of women are aware of fistulas, ranging from a low of 13% to a high of 57% in The Gambia and Sierra Leone, respectively. Marital status, age, media and internet use, level of education, sexual activity, employment and health knowledge were all discovered to be significant factors in shaping a woman’s awareness of obstetric fistulas.

**Conclusion:**

Increasing efforts to improve educational attainment, media access, workplace opportunities for women and health knowledge may have the potential to further increase fistula awareness in these and neighbouring countries. Addressing inadequate maternal health care and increasing women’s rights in these countries can reduce the rates of childbirth injuries like obstetric fistulas.

**Contribution:**

This article provides insights to the importance of targeting awareness initiatives through education, media, and community engagement, particularly among women who are younger, less educated, or socially marginalised.

## Introduction

Obstetric fistulas represent a devastating and often overlooked consequence of inadequate maternal health care. Obstetric fistulas are childbirth injuries that result from prolonged obstructed labour, which compresses the mother’s soft tissues between the baby’s head and the bony pelvis, leading to a hole or fistula formation between the vagina and nearby organs such as the bladder or rectum.^1^ Urine and/or faeces leak uncontrollably through the vagina due to this passage, resulting in severe physical discomfort, social stigma and emotional distress for the affected women.^1^ Fistulas can represent the intersection of medical disparities, gender inequality and social injustice that exists in childbirth in continents like Africa. Due to the unique features of The Gambia, Côte d’Ivoire, Madagascar and Sierra Leone – including varied awareness and prevalence of fistulas, education and women’s empowerment challenges and available nationally representative data on fistulas^2,3,4^ – these countries are useful benchmarks for understanding the state of fistulas in the region.

As of 2023, researchers estimate that two million girls and women continue to live with obstetric fistulas, while 50 000–100 000 women develop an obstetric fistula each year;^5^ but obstetric fistulas are largely preventable and treatable.^6^ Further investigation is needed to identify the socio-cultural factors, economic constraints and the lack of awareness of causes and symptoms of fistulas that enable them to occur and prevent women from seeking medical care. Understanding a population’s level of awareness of fistulas is perhaps the first step in prevention and intervention efforts as it empowers individuals and communities to recognise the signs and symptoms of obstetric fistulas early on and engage in prevention behaviours before a fistula even arises. Considering the importance of fistula awareness for both prevention and intervention, we use nationally representative Demographic and Health Survey (DHS) data from The Gambia, Sierra Leone, Côte d’Ivoire and Madagascar to (1) estimate the *prevalence* of fistulas in these countries, (2) estimate the prevalence of fistula *awareness*, and (3) understand possible socioeconomic and cultural components of fistula awareness. This research aims to increase knowledge of the importance of basic fistula awareness and propose possible pathways to further increase this awareness in the region.

### Fistula awareness

Fistula awareness includes understanding the risk factors, prevention methods and treatment approaches for fistulas. This is important for several reasons. Firstly, awareness campaigns can educate communities, health care providers and policymakers about the causes of obstetric fistulas and the importance of timely access to maternal healthcare for both prevention and treatment.^7^ Secondly, as obstetric fistulas often result in chronic incontinence, leading to many complications and psychological distress,^8^ fistula awareness can help women recognise symptoms early and seek appropriate medical care to prevent further complications and improve symptoms through treatment.^9^ Thirdly, fistula awareness can inform affected women about the availability of surgical repair procedures and rehabilitation services, helping them seek appropriate care and improve their quality of life. Ultimately, awareness is crucial for addressing the health, social and economic impacts of obstetric fistulas and ensuring that affected women receive the support, treatment and care they need to lead dignified and fulfilling lives.^10^

To combat the prevalence of fistulas, health care initiatives in many countries are raising awareness of obstetric fistula avoidance and symptoms, yet inequalities arise in who receives this information and who can utilise it as socio-cultural barriers arise, limiting access for some women while enabling it for others.^11^ Numerous women, particularly those in the most marginalised and disadvantaged circumstances, lack access to the essential resources that help prevent and effectively treat fistulas.^12^ For example, research suggests that age and marital status impact access to fistula resources and awareness. Younger women may have less access to information about fistulas and their prevention, as they may be less likely to receive comprehensive sexual and reproductive health education.^13^ Conversely, older women who have already experienced childbirth may be more aware of fistulas due to personal experience or exposure to information through health care services, community outreach programmes or word of mouth.^14^ Additionally, women with children may have better access to fistula awareness through antenatal care services provided during pregnancy, where health care providers can educate them about the risks of fistulas.^15^ However, the situation may differ for unmarried sexually active women, who might encounter limited or stigma-affected access to health care services. This lack of access could significantly impact their awareness of fistulas.^16^

### Country differences in fistula awareness

Countries vary significantly in their fistula prevalence and awareness. Existing research suggests that in sub-Saharan Africa, religious teachings, wealth and media usage may influence fistula awareness as these factors also shape attitudes towards reproductive health, childbirth and maternal care.^17^ In certain countries like The Gambia and Madagascar, religious teachings on childbirth may emphasise the sanctity of childbirth and motherhood, which could potentially lead to a lack of acknowledgement of childbirth complications like fistulas.^18^ Conversely, women in Sierra Leone who gained knowledge about childbearing and family planning through media exposure – listening to the radio and reading newspapers – were at higher odds of fistula awareness.^19^ Full access to technology enables women to make empowered health decisions.^19^ For example, mobile phones assist women, especially those in rural areas or with restricted mobility, to access vital information and services. Access to mobile phones can facilitate women’s access to health information, telemedicine services, appointment scheduling and medication management contributing to better health outcomes and overall empowerment.^20^

It is important to note, however, that many of the women who have access to media are in higher wealth brackets and are less likely to experience fistulas. Research suggests that women who experience fistulas are often poorer and less educated, which further limits their access to resources that improve fistula awareness and treatment,^21^ making it particularly important to intentionally target specific groups with fistula education. Lastly, increases in women’s access to education in these countries have been linked to increases in awareness surrounding obstetric fistulas.^22^ Formal education often equips individuals with basic health literacy skills, incorporating them into a space that may offer information about health, ideally including obstetric fistulas, their causes, symptoms and prevention measures.^23^

Research links fistula awareness to decreases in fistula prevalence, increases in fistula detection and increases in knowledge of surgical repair techniques. Furthermore, fistula and other health care awareness have been associated with increased education and media usage.^4^ Considering the importance of fistula awareness and the existing knowledge that this awareness can be influenced by socioeconomic factors, we aim to add to this conversation by examining the prevalence of fistula awareness in The Gambia, Sierra Leone, Côte d’Ivoire, and Madagascar, as well as gain a more robust understanding of socioeconomic factors related to this awareness.

## Research methods and design

### Data and methods

The cross-sectional data used in this analysis come from DHS, a comprehensive, internationally recognised project that collects and disseminates data on demographic and health indicators in over 90 countries around the world. The DHS programme collects data from representative samples of households, generally focused on women of reproductive age (15–49 years). For this study, we examine the most recent DHS data from The Gambia, Sierra Leone, Côte d’Ivoire and Madagascar.

**FIGURE 1:**
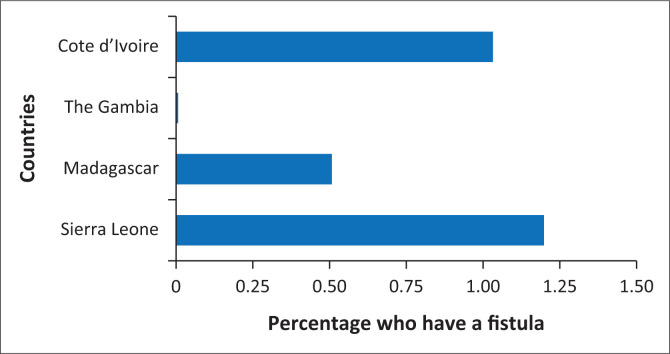
Women who have fistula by country.

**FIGURE 2:**
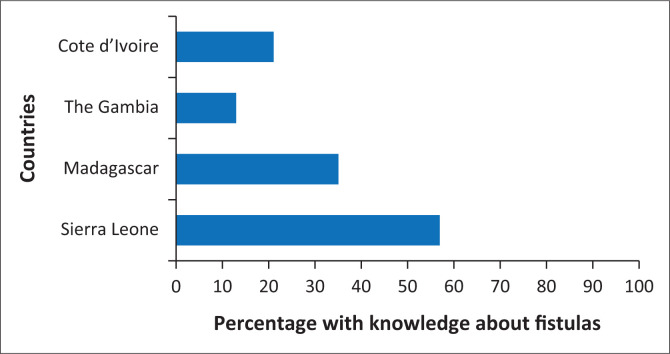
Knowledge of fistulas by country.

We selected these countries for their presence in sub-Saharan Africa where fistulas are most common, their regional variability (The Gambia, Sierra Leone, and Côte d’Ivoire in West Africa and Madagascar in East Africa), their recent survey availability that includes fistula questions (The Gambia and Sierra Leone in 2019–2020 and Côte d’Ivoire and Madagascar in 2021) and lastly, their diverse experiences with both fistula prevalence and fistula awareness (see Figure 1 and Figure 2). Missing data were below 1% for all variables included in the analysis; therefore, listwise deletion was employed, as the negligible level of missingness was unlikely to introduce bias or meaningfully reduce statistical power (The initial combined sample size across all surveys was *n* = 61 185 women aged 15–49 years. Of these, *n* = 60 810 women were asked about fistula awareness. After including all controls in the model, the final analytic sample was *n* = 60 128). We found no substantial differences when attempting alternative strategies for handling missing data. Model diagnostics indicated no concerns with multicollinearity, as all variance inflation factors (VIFs) were below conventional thresholds. Sensitivity analyses produced consistent results, suggesting the robustness of the findings. Institutional Review Board (IRB) approval was not required for the use of this secondary, publicly accessible data, although it is important to note that DHS enumerators obtain informed consent and follow ethical standards.

### Measures

Our key variables of interest are fistula awareness and fistula prevalence. For fistula *awareness*, women were asked if they have heard about fistulas, and for fistula *prevalence*, women were asked if they had urinary or faecal discharge through the vagina; both are dichotomous with response options: yes (coded 1) and no (coded 0).

Control measures include urban or rural residence, current relationship status, current age, wealth, educational attainment, justification for wife beating (composite indicator that measures whether respondents believe a husband is justified in hitting or beating his wife under several specified circumstances), number of births, media usage, internet usage, pregnancy termination, sexual activity, employment status, health knowledge (composite measure assessing women’s awareness of key health topics) and religious affiliation. For details about the measures and coding procedures, see Table 1.

**TABLE 1:** Description of measures and coding strategies used in this study.

Variable	Question	Coding
**Focal variables**		
Fistula awareness	Has the respondent ever heard about fistula?	0 = No 1 = Yes
Fistula prevalence	Has the respondent had urinary or faecal discharge through the vagina?	0 = No 1 = Yes
**Controls**		
Urban	If the respondent lives in an urban or rural location	0 = Rural area 1 = Urban area
Relationship status	Respondent’s current relationship status	0 = Never married 1 = Married 2 = Cohabitating 3 = Widowed, divorced or separated
Age	A self-report of respondent’s age at the time of survey	Ranges from 15 to 49 years old
Wealth	A single, composite score calculated by combining information about a household’s ownership of various assets like televisions, vehicles and quality of housing materials, as well as access to water and sanitation, creating a measure of their relative economic standing based on the Demographic and Health Survey (DHS) data	Ranges from -2.1 to 4.1
Education in years	A self-report of respondent’s number of years of completed education at the time of survey	Ranges from 0 to 20 years of education
Justified wife beating	Four dichotomous questions were asked of the respondent, whether wife beating was considered justified in the following hypothetical situations: (1) when the wife goes out without telling her husband, (2) wife neglects the children, (3) wife argues with her husband and (4) wife burns the food	Each question is coded: 0 = no justification for wife beating 1 = yes, justification for wife beating We added these together to create a numerical scale ranging from 0 to 4 with higher numbers representing higher justification for wife beating
Total number of births	A self-reported count of the number of children the respondent has given birth to	Ranges from 0 to 21 births
Media usage	Women were asked three questions: (1) if they read the newspaper or magazine, (2) if they listen to the radio or (3) if they watch television	Each question is coded 0 = not at all 1 = less than once a week 2 = at least once a week We added these together such that a value of 0 corresponds to never utilising any media and a score of 6 corresponds to using all three forms of media at least once a week.
Ever used internet	Has the respondent ever used the internet?	0 = No 1 = Yes
Terminated pregnancy	Has the respondent ever terminated a pregnancy?	0 = No 1 = Yes
Sexual activity	What is the respondent’s sexual history?	0 = Never had sex 1 = Not sexually active in last 4 weeks 2 = Sexually active in last 4 weeks
Currently employed	Is the respondent currently employed?	0 = Not working 1 = Employed
Health knowledge	Women were asked four questions about their knowledge about various health components: (1) knowledge about timing of an ovulatory cycle, (2) knowledge of any contraceptive method, (3) knowledge of oral rehydration and (4) knowledge of a sexually transmitted infection and AIDS.	Each question is coded 0 = No knowledge 1 = Yes, knowledge We added these together to produce a health knowledge scale ranging from 0 (no knowledge of these things) to 4 (knowledge of all four items).
Religious affiliation	What is the respondent’s religious affiliation?	1 = Muslim 2 = Catholic 3 = Evangelical or Christian 4 = ‘Other’

### Analytic strategy

Figure 1 shows the percentage of women who have a fistula by country. Figure 2 shows the percentage of women with knowledge and/or awareness of fistulas by country. Table 2 shows the weighted descriptive statistics for our sample overall and by country. Table 3 shows the results of weighted logistic regression models of fistula knowledge overall and by country. Results in Table 3 are presented as odds ratios (OR). Weighted descriptive statistics were used in this analysis to ensure representativeness as not all women were asked about fistulas.

**TABLE 2:** Weighted percent and mean descriptive statistics overall and by country.

Variable	Min	Max	Combined countries			Côte d’Ivoire			The Gambia			Madagascar			Sierra Leone		
			%	Mean	s.e.	%	Mean	s.e.	%	Mean	s.e.	%	Mean	s.e.	%	Mean	s.e.
Has a fistula	0.0	1.0	0.01	-	-	0.01	-	-	0.00	-	-	0.01	-	-	1.2	-	-
Knowledge of fistulas	0.0	1.0	32.90	-	-	20.90	-	-	12.90	-	-	35.10	-	-	57.0	-	-
Urban	0.0	1.0	47.60	-	-	60.80	-	-	73.70	-	-	22.20	-	-	41.1	-	-
Relationship status	0.0	3.0	-	-	-	-	-	-	-	-	-	-	-	-	-	-	-
Never married (reference)	-	-	30.10	-	-	33.10	-	-	31.20	-	-	25.00	-	-	32.5	-	-
Married	-	-	51.40	-	-	34.00	-	-	63.20	-	-	51.90	-	-	58.5	-	-
Cohabitating	-	-	10.80	-	-	27.50	-	-	0.10	-	-	10.10	-	-	3.9	-	-
Wid, div or separated	-	-	7.70	-	-	5.40	-	-	5.40	-	-	13.00	-	-	5.1	-	-
Age	15.0	49.0	-	28.5	0.05	-	28.6	0.10	-	28.1	0.11	-	28.5	0.08	-	28.7	0.09
Wealth	−2.1	4.1	-	0.2	0.01	-	0.3	0.01	-	0.3	0.01	-	0.1	0.01	-	0.2	0.01
Education in years	0.0	20.0	-	4.5	0.02	-	4.2	0.06	-	5.7	0.06	-	5.2	0.03	-	4.6	0.04
Justified wife beating	0.0	4.0	-	0.9	0.01	-	0.6	0.01	-	1.0	0.01	-	0.7	0.01	-	1.3	0.01
Total number of births	0.0	21.0	-	2.5	0.01	-	2.4	0.04	-	2.4	0.03	-	2.5	0.02	-	2.6	0.02
Media usage	0.0	6.0	-	1.8	0.01	-	1.9	0.02	-	2.7	0.01	-	1.6	0.01	-	1.2	0.01
Ever used internet	0.0	1.0	28.70	-	-	31.70	-	-	65.60	-	-	14.90	-	-	14.4	-	-
Ever terminated pregnancy	0.0	1.0	14.30	-	-	19.40	-	-	16.80	-	-	13.50	-	-	8.5	-	-
Sexual activity	0.0	2.0	-	-	-		-	-		-	-		-	-		-	-
Never had sex (reference)	-	-	14.50	-	-	12.00	-	-	28.60	-	-	11.10	-	-	10.3	-	-
Not active in last 4 weeks	-	-	32.30	-	-	32.80	-	-	30.10	-	-	28.50	-	-	38.2	-	-
Active in last 4 weeks	-	-	53.20	-	-	55.20	-	-	41.30	-	-	60.40	-	-	51.5	-	-
Currently employed	0.0	1.0	68.60	-	-	58.20	-	-	59.80	-	-	80.10	-	-	71.6	-	-
Health knowledge	0.0	4.0	-	3.1	0.00	-	3.1	0.01		3.1	0.01	-	3.1	0.01	-	3.2	0.01
Religious affiliation	0.0	3.0	-	-	-	-	-	-	-	-	-	-	-	-		-	-
Muslim (reference)	-	-	45.40	-	-	44.50	-	-	96.40	-	-	32.50	-	-	23.2		
Catholic	-	-	35.10	-	-	17.50	-	-	3.50	-	-	34.60	-	-	76.7	-	-
Methodist	-	-	14.10	-	-	29.20	-	-	0.00	-	-	22.70	-	-	0.0		
Other	-	-	5.30	-	-	8.90	-	-	0.00	-	-	10.30	-	-	0.0	-	-

Note: Combined countries: 61 185; Côte d’Ivoire: 14 877; The Gambia: 11 865; Madagascar: 18 869; Sierra Leone: 15 408. Linearised standard error, presented with mean values.

s.e., standard error.

The DHS programme recommends using the women’s sample weight (variable v005) to create representative population estimates by accounting for unequal selection probabilities and non-response when the unit of analysis is individual women. Model fit was statistically significant for the overall and country-specific models, indicating that the set of predictors jointly explained significant variation in the outcome. Given the survey design, pseudo R^2^ statistics were not reported; instead, design-based Wald tests were used to assess model adequacy. Table 4 reports confidence intervals for the odds ratios presented in Table 3. All analyses were conducted using Stata 18.0 (StataCorp LLC, College Station, TX).

**TABLE 3:** Weighted logistic regression models of fistula knowledge by country.

Variable	Combined countries	Côte d’Ivoire	The Gambia	Madagascar	Sierra Leone
Urban	1.02	1.17*	0.78*	1.09	0.97
**Relationship status**					
Married	1.23***	1.17	1.66**	1.06	1.31***
Cohabitating	1.29***	1.16	1.06	1.39***	1.33*
Wid, Div or Separated	1.17**	1.13	1.14	1.14	1.71***
Age	1.03***	1.03***	1.02***	1.02***	1.03***
Wealth	0.93***	1.02	1.17*	0.86***	0.89**
Education in years	1.04***	1.04***	1.05***	1.05***	1.04***
Justified wife beating	1.06***	1.10***	1.10***	1.03	1.04***
Total number of births	1.00	1.02	1.01	0.98	1.03**
Media usage	1.14***	1.19***	1.07*	1.17***	1.07***
Ever used internet	1.21***	1.16	1.02	1.08	1.34***
Terminated pregnancy	1.08*	1.16*	1.19*	0.86**	1.34***
**Sexual activity**					
Not active in last 4 weeks	1.36***	2.19***	0.97	1.04	1.81***
Active in last 4 weeks	1.34***	2.40***	0.87	1.14	1.71***
Currently employed	1.26***	1.32***	1.45***	1.03	1.25***
Health knowledge	1.64***	1.50***	1.31***	1.52***	1.88***
**Religious affiliation**					
Catholic	0.99	1.00	1.47*	0.93	0.92
Methodist	0.80***	1.15	-	0.54***	1.01
Other	0.91	0.85	3.66	0.93	-
**Country**					
The Gambia	0.45	-	-	-	-
Madagascar	2.12	-	-	-	-
Sierra Leone	5.59	-	-	-	-

Note: Combined countries: 59 751; Côte d’Ivoire: 14 592; The Gambia: 11 654; Madagascar: 18 355; Sierra Leone: 15 193. Reference categories: never married; never had sex, Muslim, Côte d’Ivoire.

*** , *p* < 0.001;

** , *p* < 0.01;

* , *p* < 0.05.

## Results

Figure 1 and Figure 2 show the percentage of women who have a fistula by country and the percentage of women with knowledge about a fistula by country, respectively. Sierra Leone has the largest number of women with a fistula (Figure 1) and the highest percentage with knowledge about fistulas (Figure 2). Conversely, The Gambia has the lowest fistula prevalence (Figure 1) as well as fistula knowledge (Figure 2). It is logical that the country with the lowest reported prevalence of fistulas also demonstrates the lowest levels of fistula awareness and vice versa, as lower exposure to the condition may limit public knowledge and recognition. Understanding this relationship is important because it highlights how awareness efforts should be tailored to each country’s epidemiological context, ensuring that prevention and education initiatives are targeted where and how they are most needed.

Table 2 shows the descriptive statistics for our DHS sample overall and by country. Overall, 0.01% of respondents report having a fistula, which ranges from 0% in The Gambia up to 1.2% in Sierra Leone. Regarding knowledge of fistulas, across all countries, roughly 33% of women reported having such knowledge. However, there is substantial cross-national variation in fistula awareness, ranging from 13% of women in The Gambia to 57% in Sierra Leone.

Overall, 48% of women live in an urban area, and 30% have never been married, 51% are currently married, 10% are cohabitating and roughly 8% are widowed, divorced or separated. On average, women are 28.5 years old, and the wealth index is, on average, 0.2. Women have an average of 4.5 years of completed education ranging from 4.2 years in Côte d’Ivoire to 5.7 in The Gambia. Women average a total of 2.5 births, and roughly 14% of women have terminated a pregnancy overall, ranging from 8% in Sierra Leone up to 19% in Côte d’Ivoire (see Table 2 for additional descriptive statistics of the variables analysed in this study).

Table 3 shows the results of weighted logistic regression models on fistula awareness overall and by country. Overall, our analysis found no significant difference in fistula awareness between women in urban versus rural areas. Conversely, living in an urban area varied in its effects from country to country, increasing the odds of fistula knowledge by 1.17 times (*p* = 0.03) in Côte d’Ivoire and reducing the odds of fistula knowledge by 0.78 times (*p* = 0.02) in The Gambia. Overall, women who are married, cohabiting, widowed, divorced or separated have increased odds of being knowledgeable about fistulas compared to never-married women. This is also true for married women in The Gambia, cohabitating women in Madagascar, and married, cohabitating and widowed, divorced or separated women in Sierra Leone. All other relationship statuses among these four countries are not statistically different from never-married women.

The odds of knowing about fistulas grow about 1.02 times for each additional year of age in The Gambia (*p* = 0.001) and Madagascar (*p* < 0.001) and by 1.03 times in Sierra Leone (*p* < 0.001) and Côte d’Ivoire (*p* < 0.001). The odds ratio for age remains generally consistent across all four countries, suggesting a slightly greater likelihood of fistula awareness as age increases. For example, if two women differ by 2 years of age, the older woman has predicted odds of knowing about a fistula that are 1.06 (1.03*1.03) times larger than the younger woman. If two women differ in age by 10 years, the odds that the older woman would know about a fistula are 1.34 times larger than those of the younger woman. Similarly, the odds of knowing about fistulas are predicted to grow about 1.04 times (*p* < 0.001) larger for each additional year of education. These results generally remain consistent across all four countries, rising by 1.04 times in Côte d’Ivoire (*p* < 0.001) and Sierra Leone (*p* < 0.001) and by 1.05 times in The Gambia (*p* < 0.001) and Madagascar (*p* < 0.001).

Overall, wealth had a negative association with fistula knowledge. By country, women who were wealthier had decreased knowledge of fistulas in Madagascar (OR = 0.86, *p* = 0.001) and Sierra Leone (OR = 0.89, *p* = 0.001). Conversely, wealthier women had increased knowledge of fistulas in The Gambia (OR = 1.17, *p* = 0.001). There was no statistically significant relationship between wealth and fistula knowledge in Côte d’Ivoire. Women in all four countries who had higher justification for wife beating were more likely to be knowledgeable about fistulas compared to those who had lower justification. More specifically, women with increased justification for wife beating had odds of having fistula knowledge ranging from 1.03 times (*p* = not significant) more likely in Madagascar to 1.10 times more likely in Côte d’Ivoire (*p* < 0.001) and The Gambia (*p* = 0.001). No significant difference in fistula knowledge was found among women with different numbers of births overall and in The Gambia, Madagascar and Côte d’Ivoire. However, an increased total number of births was associated with having increased fistula awareness in Sierra Leone (OR = 1.03, *p* = 0.03). The odds of knowing about fistulas are predicted to be about 1.14 times (*p* < 0.001) larger for women with greater media usage than those with reduced media usage, holding all other variables constant. Increased media usage ranged slightly in its effects on fistula knowledge from country to country, improving the odds of fistula knowledge by 1.07 times in The Gambia (*p* = 0.03) and Sierra Leone (*p* < 0.001) up to 1.19 times in Côte d’Ivoire (*p* < 0.001).

Similarly, the odds of fistula awareness are predicted to be 1.21 times (*p* < 0.001) larger for women with greater internet usage than those with no internet usage, holding all other variables constant. However, increased internet usage varied in its effects on fistula knowledge from country to country, improving the odds of fistula knowledge by 1.34 times (*p* < 0.001) in Sierra Leone but having no statistically significant relationship in the other three countries. Overall, women who have terminated pregnancies have increased odds of being knowledgeable about fistulas, roughly 1.08 times (*p* = 0.03) greater than women who have never terminated a pregnancy. Pregnancy termination increased the odds of improved fistula knowledge in every country except Madagascar, where women who had terminated a pregnancy had lower odds (OR = 0.86 times, *p* = 0.009) of having knowledge of fistulas.

Overall, women who have been sexually inactive and women who have been sexually active in the last 4 weeks show increased odds of being knowledgeable about fistulas compared to women who have never been sexually active. This is also true for women who have not been sexually active in the last 4 weeks in Côte d’Ivoire (OR = 2.19, *p* < 0.001) and Sierra Leone (OR = 1.81, *p* < 0.001) and sexually active women in Côte d’Ivoire (OR = 2.40, *p* < 0.001) and Sierra Leone (OR = 1.71, *p* < 0.001). All other sexual activity statuses among these four countries are not statistically different from women who have never been sexually active.

Overall, women who are currently employed were found to have odds 1.26 times (*p* < 0.001) greater than those who are unemployed. More specifically, employed women had odds of having fistula knowledge ranging from 1.03 times in Madagascar (*p* = not statistically significant) to 1.45 times in The Gambia (*p* < 0.001). The odds of knowing about fistulas are predicted to be about 1.64 times (*p* < 0.001) larger for women with better scores on the health knowledge scale, holding all other variables constant. Increased health knowledge ranged in its effects on fistula knowledge by country and improved the odds of fistula knowledge by 1.31 times (*p* < 0.001) in Côte d’Ivoire up to 1.88 times (*p* < 0.001) in Sierra Leone. Overall, women who identify as Methodist had reduced odds of being knowledgeable about fistulas compared to women who are Muslim (reference category). By country, women who are Methodist had decreased knowledge of fistulas in Madagascar (OR = 0.54, *p* < 0.001). However, Catholic women had increased knowledge of fistulas in The Gambia (OR = 1.47, *p* < 0.001). All other religious statuses among these four countries were not statistically different from the Muslim women. Table 4 reports confidence intervals for the odds ratios presented in Table 3.

**TABLE 4:** 95% confidence intervals for the weighted logistic regression models of fistula knowledge by country presented in Table 3.

Variable	Combined countries		Côte d’Ivoire		The Gambia		Madagascar		Sierra Leone	
**Urban**	0.95	1.08	1.01	1.35	0.63	0.96	0.98	1.21	0.86	1.09
**Relationship status**										
Married	1.13	1.33	0.95	1.45	1.18	2.35	0.93	1.22	1.16	1.49
Cohabitating	1.16	1.43	0.95	1.42	0.22	5.23	1.17	1.66	1.06	1.66
Wid, div or separated	1.05	1.31	0.83	1.53	0.72	1.80	0.95	1.30	1.37	2.14
Age	1.02	1.03	1.02	1.04	1.01	1.04	1.02	1.03	1.02	1.03
Wealth	0.89	0.97	0.93	1.11	1.03	1.32	0.81	0.92	0.83	0.95
Education in years	1.03	1.05	1.02	1.06	1.04	1.08	1.03	1.07	1.03	1.06
Justified wife beating	1.04	1.08	1.04	1.16	1.04	1.16	0.99	1.07	1.01	1.07
Total number of births	0.99	1.02	0.98	1.05	0.97	1.06	0.95	1.00	1.00	1.06
Media usage	1.12	1.17	1.13	1.24	1.01	1.14	1.13	1.21	1.04	1.11
Ever used internet	1.12	1.31	0.99	1.38	0.85	1.21	0.94	1.23	1.15	1.56
Terminated pregnancy	1.01	1.16	1.01	1.34	1.00	1.43	0.77	0.96	1.15	1.57
**Sexual activity**										
Not active in last 4 weeks	1.23	1.51	1.59	3.01	0.68	1.39	0.89	1.22	1.54	2.14
Active in last 4 weeks	1.21	1.49	1.74	3.30	0.60	1.27	0.96	1.34	1.44	2.02
Currently employed	1.18	1.34	1.15	1.51	1.23	1.73	0.93	1.15	1.13	1.39
Health knowledge	1.59	1.70	1.39	1.62	1.11	1.55	1.45	1.60	1.77	1.99
**Religious affiliation**										
Catholic	0.93	1.05	0.83	1.19	0.99	2.19	0.85	1.02	0.84	1.02
Methodist	0.73	0.87	0.99	1.34	-	-	0.48	0.61	0.18	5.81
Other	0.82	1.02	0.68	1.05	0.09	10.50	0.82	1.06	-	-
**Country**										
The Gambia	0.40	0.50	-	-	-	-	-	-	-	-
Madagascar	1.97	2.29	-	-	-	-	-	-	-	-
Sierra Leone	5.13	6.10	-	-	-	-	-	-	-	-

Note: Reference categories: never married; never had sex, Muslim, Côte d’Ivoire.

## Discussion

Women with an obstetric fistula often endure chronic incontinence, social isolation and often face abandonment by their families and communities,^1^ making prevention and quick treatment an important goal. Using data from DHS, the current study aimed to investigate associated socioeconomic factors related to fistula awareness in The Gambia, Côte d’Ivoire, Sierra Leone and Madagascar. Overall, 33% of women in these four countries are aware of fistulas, ranging from a low of 13% to a high of 57%. Marital status, age, media use, internet use, level of education, sexual activity, employment and other health knowledge were all characteristics discovered to be significant factors in increasing a woman’s awareness of obstetric fistulas in these countries. By assessing the state of fistula prevalence and awareness in four countries in West and East Africa and examining existing efforts made by those in the public health, policy, medicine and government sectors to address fistulas, we hope to provide pathways forward for these and similar countries. For example, roughly 1.2% of respondents in Sierra Leone report having a fistula, while 57% report fistula knowledge. Sierra Leone began implementing ‘Fistula Advocacy’ programmes in which women who had previously been diagnosed and treated with obstetric fistulas helped to find women with undiagnosed fistulas and support the recovery of women still going through the process. Advocates used their personal stories to redefine their identity, change perceptions and reduce stigma surrounding obstetric fistulas. By sharing their experience, they aimed to raise awareness and dispel myths associated with the condition.^19,24,25^

Not only did Sierra Leone implement ‘Fistula Advocacy’, in 2010, the Sierra Leone Government launched the Free Health Care Initiative^26^ that enabled pregnant women, lactating mothers and infants under the age of five to access medical care services completely free of charge.^27^ Surgical repair is the primary treatment for obstetric fistulas. However, access to fistula repair services is limited in many African countries due to a shortage of trained health care providers, inadequate health care infrastructure and financial barriers. Obstetric fistulas are not merely a medical issue; rather, they represent a poignant intersection of health care, social injustice and women’s rights. The suffering endured by women with obstetric fistulas is a manifestation of gender inequality, evident in premature childbearing, malnutrition, poor physical development before childbirth and inadequate prenatal and delivery care.^6^ While this health care initiative was not directly related to fistulas, surgical treatment has been described as a crucial factor influencing recovery from physical and psychological symptoms and social inclusion,^19^ suggesting the Sierra Leone government is working to improve poor health indicators, including fistulas, in a variety of ways.^27^

In addition to explicit fistula programmes and medical services, the education sector may be an indirect way to target fistula awareness as this and other research suggest that formal education has the potential to equip individuals with basic health literacy skills, enabling them to understand and retain information about obstetric fistulas, their causes, symptoms and preventive measures.^23^ Further, education can delay marriage, sexual initiation and early pregnancy, thereby reducing young women’s biological and social vulnerability to obstetric fistulas.^22,31^ Complementing formal education with comprehensive sexual and reproductive health education can further strengthen adolescents’ ability to make informed choices, preventing early childbearing and associated complications.^13,31^

United Nations International Children’s Emergency Fund (UNICEF) reports that in August 2018, Sierra Leone introduced the Free Quality School Education (FQSE) programme. This programme has worked to reduce financial barriers to accessing education while also eliminating extra school fees throughout the year by increasing public spending on education.^26^ Sierra Leone is not alone, with Côte d’Ivoire prioritising the improvement of education access and quality as well as mandating compulsory education from ages 6 to 16.^28^ While these investments in education could aid in increasing access to fistula knowledge, they could also lead to increased opportunities for women in the workplace, which, as this and other research show, is also related to fistula awareness.^29^ As women increase participation in various institutions, they can contribute to and learn from discussions about health-related matters that often occur between women in these spaces. These conversations can raise awareness about obstetric fistulas, symptoms, prevention and treatments.^30^ As access to health-related information has been shown to be closely related to health outcomes for women in sub-Saharan Africa,^31^ creating educational opportunities for women can enhance health knowledge and improve overall health outcomes.

Lastly, educated and/or working women often serve as role models within their communities. If women can utilise this authority and their increased awareness and understanding of obstetric fistulas, there is potential for a ripple effect as they share that information with family members, friends and neighbours.^32^ Researchers have investigated the impact of adult literacy and skill acquisition programmes on women’s empowerment and self-reliance in The Gambia, with findings suggesting that these programmes positively influence women’s ability to access education and employment opportunities.^33^ By addressing gender disparities in education and promoting skill development, the project aims to empower women and enhance their participation in the workforce, contributing to broader efforts to advance gender equality and socioeconomic development in The Gambia.

Considering these direct and indirect efforts to improve fistula awareness – creating fistula programmes, expanding medical coverage, improving educational attainment and increasing women’s empowerment through skill building – we hope to see further improvements in fistula awareness. Future research should examine the influence of these changes on increases in fistula awareness to ensure all women can access this important information and that these direct and indirect efforts are, in fact, increasing fistula awareness and thus decreasing fistula prevalence.

### Limitations

Due to cultural stigma and a lack of knowledge surrounding obstetric fistulas, the accuracy of responses and honesty of reporting of fistulas may be hindered. Consequently, self-reported awareness of fistula may be underestimated due to stigma or misunderstanding, which could bias prevalence estimates downward. This is unfortunate because an accurate understanding of the prevalence of fistulas is key to getting the prevention and corrective resources to the women who need them. Further, using a single-item measure for fistula awareness may limit the depth and reliability of the construct captured, potentially introducing measurement bias and affecting the interpretation of the respondent’s true levels of awareness. To acquire a more complete understanding of fistula awareness and prevalence in these countries, a mixed-methods research approach should be undertaken to ask multiple detailed questions about fistula prevalence and awareness, such as what their knowledge of fistulas is, to assess accuracy and potential gaps in awareness.

Further, the cross-sectional nature of the DHS data limits our ability to infer causality. Additionally, study findings are only reflective of phenomena in The Gambia, Sierra Leone, Côte d’Ivoire and Madagascar and are not generalisable to all West and East Africa. Lastly, the policy recommendations for improving fistula awareness were specifically tailored to the unique contexts of the countries included in this study. Because of this, these recommendations may not necessarily be effective across all sub-Saharan African countries.

## Conclusion

Obstetric fistulas remain both a profound medical challenge and a marker of persistent gender and socioeconomic inequalities in health care. The findings of this study underscore the importance of targeting awareness initiatives through education, media and community engagement, particularly among women who are younger, less educated or socially marginalised. Beyond identifying correlates of awareness, these results emphasise that improving awareness is not only a medical task but also a matter of social justice, as it intersects with women’s empowerment, access to resources and broader public health priorities.

To make sustainable progress, these and other countries must build on existing advocacy and education programmes while strengthening health systems to ensure timely prevention and treatment of fistulas. Investing in women’s education, expanding access to health information and reducing barriers to care can collectively reduce the burden of fistula and improve the quality of life for affected women and girls. Addressing fistula awareness in this way is not simply about increasing knowledge; it is about dismantling systemic barriers that perpetuate gendered health disparities.
